# P11 An unusual case of Systemic Sclerosis/JDM overlap in a child with PAX8-related congenital hypothyroidism

**DOI:** 10.1093/rap/rkac067.011

**Published:** 2022-09-28

**Authors:** Rachel Smith, Rebecca Ward, Samundeeswari Deepak, Satyapal Rangaraj, Kishore Warrier

**Affiliations:** Nottingham University Hospitals, Nottingham, United Kingdom; Nottingham University Hospitals, Nottingham, United Kingdom; Nottingham University Hospitals, Nottingham, United Kingdom; Nottingham University Hospitals, Nottingham, United Kingdom; Nottingham University Hospitals, Nottingham, United Kingdom

## Abstract

**Introduction/Background:**

Juvenile dermatomyositis is a rare inflammatory disease with a reported incidence of 0.8–4.1 per million children per year. It presents with a constellation of symptoms and signs of inflammation of skin and muscle, and is associated with significant morbidity if untreated. Juvenile systemic sclerosis (JSSc) is an even rarer rheumatological condition of childhood with significant risk of internal organ involvement, especially cardiopulmonary disease. We herewith present the case of a 7-year-old boy with history of hypothyroidism associated with a PAX8 gene variant who presented with clinical features of an overlap of Systemic Sclerosis with JDM with supportive autoantibody profile.

**Description/Method:**

A 7-year-old boy presented with a history of restricted range of movements in his hands – noted due to difficulties with pen grip – and calcinosis. He reported symptoms of fatigue, intolerance to cold with colour changes to hands and feet, stiffness of movements particularly in the morning, and infrequent episodes of choking. His past medical history was significant for hypothyroidism secondary to a paternally inherited PAX8 missense mutation, and there is a family history of Raynaud’s and hypothyroidism. A detailed examination identified diffusely swollen hands with sclerodactyly, calcinosis of his elbows and fingertips, and limited flexion of his fingers and wrists. He also had telangiectasia of his upper eyelids, Gottron’s papules over his knees and elbows, and sluggish circulation in his hands and feet. He had evidence of mild proximal muscle weakness.

His investigations revealed mild elevation of muscle enzymes with normal inflammatory markers. His autoantibody profile showed a moderately positive ANA titre of 1600 with a nucleolar pattern, and was positive for anti-NXP2 and anti-SCL-100 antibodies MRI scan of the pelvis showed no evidence of myositis, echocardiogram was normal and a high-resolution CT scan of chest was normal with no features of interstitial lung disease.

The overall picture was one of Systemic Sclerosis/JDM overlap with CREST syndrome. He was commenced on induction therapy with IV Methylprednisolone and Cyclophosphamide in view of the calcinosis. After three cycles of Cyclophosphamide and weaning course of oral corticosteroids, his energy levels are significantly improved and he has better overall mobility with no significant proximal muscle weakness (Childhood Myositis Assessment Scale score 49/53). However, flexion of his fingers remains limited albeit better; he has ongoing fingertip calcinosis but fewer lesions over his elbows. He has been commenced on weekly subcutaneous Methotrexate as he completes Cyclophosphamide.

**Discussion/Results:**

This patient had an unusual medical history of early onset thyroid dysfunction, the genetic testing for which revealed a missense PAX8 gene variant [c.49G>Ap.(Gly17Arg)] (also present in his father, who is euthyroid). This was classified as being of uncertain significance, although felt to clinically be the most likely cause for his hypothyroidism. There was no evidence to suggest that PAX8 gene mutations increased the risk of autoimmune conditions. There is evidence that PAX8 gene mutations, when expressed on malignant cells, may confer resistance to chemotherapy, which made us rethink the plan to commence him on Cyclophosphamide. However, we were reassured that in constitutional variants, there is no increased risk of cancer, and no known history of resistance to Cyclophosphamide.

This patient also demonstrated an interesting antibody pattern, as he was positive for anti-NXP2 antibodies (indicating high risk for calcinosis) and anti-SCL-100 antibodies, which are associated with a clinical subset of patients with overlap of Systemic Sclerosis and JDM and associated with extensive extra-muscular features. He had two distinct clinical phenotypes of calcinosis, which is probably explained by the presence of two autoantibodies that increase the risk of calcinosis.

**Key learning points/Conclusion:**

Although JDM has bimodal age group for presentation in childhood, only 3% of systemic sclerosis are seen in childhood, which makes this an extremely unusual presentation, given the age, gender and the overlap features (clinical and immunological).

We explored whether this rare presentation could be linked to the underlying PAX8 gene mutations, but we could not find any association.

With the clinical diagnosis of systemic sclerosis, it was important to rule out cardiopulmonary complications.

The other consideration was regarding the choice of therapeutic agent - whether the germline PAX8 gene variants could increase the risk of malignancies or cause resistance to chemotherapeutic agents like Cyclophosphamide, for neither of which we could find any evidence.

Extensive calcinosis cutis in this patient could be explained by the presence of two risk factors (CREST syndrome and JDM with NXP2 antibodies).

